# Reaction of N‐Ferrocenylcarbamates with Nitric Oxide: An Application for Detection of Inflammatory Sites In Vivo

**DOI:** 10.1002/cmdc.202500356

**Published:** 2025-08-22

**Authors:** Roman Selin, Hülya Gizem Özkan, Galyna Bila, Rostyslav Bilyy, Andriy Mokhir

**Affiliations:** ^1^ Department of Chemistry and Pharmacy Friedrich‐Alexander‐University of Erlangen Nuremberg (FAU) Organic Chemistry II 91058 Erlangen Germany; ^2^ Danylo Halytsky Lviv National Medical University Pekarska str.69 79010 Lviv Ukraine

**Keywords:** aminoferrocene, fluorogenic, inflammation, intracellular reactions, nitric oxide

## Abstract

Electron‐deficient aminoferrocenes (edAFs) exhibit anticancer activity both in vitro and in vivo. However, their mechanism of action remains unclear. Studies using fluorogenic edAF derivatives suggest that the ferrocenyl moiety undergoes oxidation or decomposition within cells, resulting in the formation of unknown products. Interestingly, this process is not significantly facilitated by H_2_O_2_, indicating that this intracellular oxidant does not alter edAFs in the cellular environment. To identify alternative endogenous oxidants, NO is investigated as a potential candidate. Under aerobic conditions, NO is found to efficiently induce the oxidation and decomposition of edAFs. This transformation is mediated by an electrophilic nitrosation reaction, followed by nitroso‐oxime tautomerism and subsequent degradation of the ferrocenyl moiety with the release of ligand‐derived oxime **7** and iron ions. These findings suggest that NO may play a key role in the intracellular modification of edAFs, potentially contributing to their anticancer activity or their metabolism or both. Building on this mechanism, an effective probe is developed for detecting NO in living cells and identifying sites of inflammation in vivo. These probes are based on a modular design that enables facile substitution of the fluorescent dye, allowing straightforward customization for diverse applications both in cellulo and in vivo.

## Graphical abstract

Nitric oxide induces decomposition of electron‐deficient aminoferrocene drugs **4** with formation of oxime **7**, which is derived from the upper cyclopentadienyl ligand. Drugs **4** are not responsive to hydrogen peroxide. Based on the latter reaction, the fluorogenic probes are developed for detecting NO in cells and inflammation in vivo.

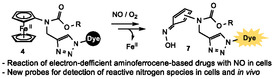

## 1 Introduction

**Figure 1:**
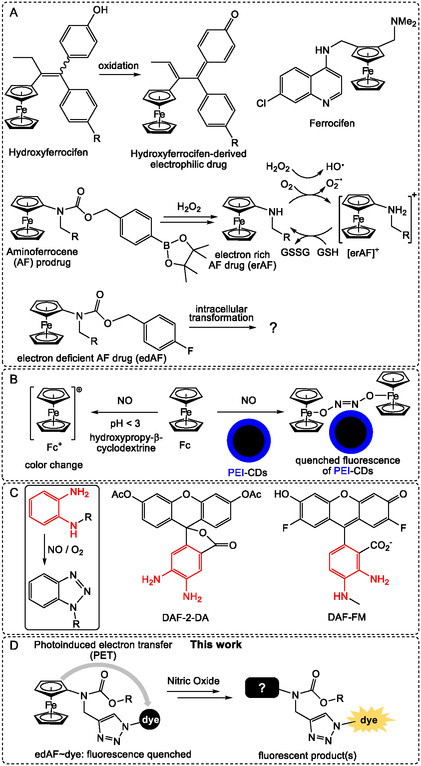
A) Examples of ferrocene derivatives, which are currently developed as drugs or prodrugs. AF prodrug reacts with H_2_O_2_ in a multistep process as described elsewhere.^[9]^ B) Known reactions of ferrocene with nitric oxide. C) The reaction of 1,2‐phenylenediamine derivatives with nitric oxide in the presence of oxygen, as well as examples of known NO probes containing a 1,2‐phenylenediamine fragment: DAF‐2‐DA and DAF‐FM. D) The investigation in this work of the reaction of NO with conjugates of edAF and fluorescent dyes (edAF–dyes) leading to fluorescent products.

Ferrocene (Fc) is a stable 18‐electron organometallic compound with chemical and physical properties similar to electron‐rich benzene derivatives.^[^ ^1^ ^]^ As a result, the Fc moiety is often used as a bioisostere for phenyl fragments to modulate drug properties. A notable example is hydroxyferrocifen (HFc, **Figure** **1**A), an organometallic analog of the active drug released from the clinically used anticancer prodrug tamoxifen. HFc is currently undergoing preclinical investigation.^[^ ^2^, ^3^, ^4^ ^]^ In cells, HFc and its analogs undergo oxidation, forming electrophilic species that modify and inhibit nucleophilic biomolecules, ultimately leading to cancer cell death. Other related examples have been recently reviewed.^[^ ^3^ ^,^ ^5^ ^]^ The incorporation of an Fc moiety into existing drugs can also help overcome drug resistance, as demonstrated by ferroquine (FQ), an analog of the antimalarial drug chloroquine (Figure 1A). The safety and efficacy of FQ in combination with artesunate were evaluated in a phase 2a clinical trial.^[^ ^6^ ^]^ In the human body, FQ is converted into its active metabolite, N‐desmethylferroquine (DMFQ) by various cytochromes.^[^ ^7^ ^]^ Notably, this transformation does not affect the Fc core. Ferrocene derivatives can also exhibit anticancer activity by generating reactive oxygen species (ROS) in cells, a consequence of the relatively low oxidation potential of Fc. For instance, aminoferrocene (AF) prodrugs developed by the group of Mokhir are converted in cancer cells into electron‐rich AF drugs (erAFs), which catalyze the conversion of oxygen to superoxide anion radicals (O_2_^·−^) and hydrogen peroxide to hydroxyl radicals (HO·) (Figure 1A). These toxic species induce cancer cell death.^[^ ^8^, ^9^, ^10^, ^11^, ^12^, ^13^, ^14^ ^]^ During the latter reaction, erAFs are oxidized to ferrocenium species [erAF]^+^, which can further decompose, releasing iron ions. In the above examples, ferrocene‐containing drugs/prodrugs are modified in cells by intracellular oxidants, generating active products. Further, appending ferrocene and AF to modulators of ferroprosis can increase the efficacy of the latter compounds.^[^ ^15^ ^,^ ^16^ ^]^

We recently reported on electron‐deficient anticancer AF drugs (edAFs) drugs^[^ ^17^ ^]^ (Figure 1A), which exhibit increased stability against oxidation, particularly from O_2_and H_2_O_2_.^[^ ^8^ ^,^ ^17^ ^]^ The mechanism of action of edAF remains unclear. In our preliminary studies, we observed a gradual increase in fluorescence of human ovarian cancer A2780 cells preloaded with fluorogenic edAF–dye conjugates and incubated for 2–4 h.^[^ ^17^ ^]^ Since edAF–dye fluorescence is quenched via photoinduced electron transfer (PET) from the ferrocenyl moiety to the dye, this suggests oxidation or degradation of the ferrocenyl group. However, fluorescence increase in edAF–dye‐preloaded cells is not accelerated by H_2_O_2_pre‐treatment, indicating that a different intracellular oxidant is responsible for the transformation of edAFs.

In our search for endogenous oxidants capable of reacting with edAFs in cells, we focused on nitric oxide (NO). As a key signaling molecule, NO plays a crucial role in regulating the cardiovascular system and neurotransmission.^[^ ^18^ ^,^ ^19^ ^]^ It is also involved in macrophage‐mediated immunity,^[^ ^20^ ^]^ as well as in tumor development and suppression.^[^ ^21^ ^]^ Notably, cancer cells produce NO,^[^ ^22^ ^]^ and macrophages in the tumor microenvironment further contribute to intracellular NO accumulation.^[^ ^23^ ^]^ Thus, NO should be available in cancer cells to react with edAF drugs. While reports on the reaction between NO and ferrocenes in living cells are absent, some evidence from cell‐free experiments suggests its feasibility. For instance, Priya et al. reported that Fc in a supramolecular complex Fc / hydroxypropyl‐*β*‐cyclodextrin (*β*‐CD) undergoes oxidation by NO, forming ferrocenium species Fc^+^/*β*‐CD (Figure 1B).^[^ ^24^ ^]^ Additionally, the same group demonstrated that in the presence of polyethyleneimine‐modified carbon dots (PEI‐cd's), Fc is converted to Fc^+^—ON=NO—Fc^+^ in the presence of NO (Figure 1B).^[^ ^25^ ^]^ Fc^+^‐ON=NO‐Fc^+^^+^ quenches PEI‐cd's fluorescence—allowing real‐time monitoring of NO via fluorescence spectroscopy. Both studies were conducted in cell‐free settings and at nonphysiological acidic conditions (pH = 3) with the goal of developing spectroscopic methods of detection of NO.

Providing that edAFs efficiently react with NO, their fluorogenic conjugated edAF–dyes can be potentially used as probes for intracellular NO. Though few fluorogenic probes for the detection of NO are known and some of them are commercially available (Figure 1C), they have important disadvantages. For example, the commercially available 1,2‐diaminobenzene‐based probes, DAF‐2‐DA and DAF‐FM, are unstable under oxidative conditions. Therefore, NO monitoring using these probes requires the careful selection of controls, which makes the measurements more complicated.^[^ ^26^ ^,^ ^27^ ^]^ This justifies studies toward improved probes, based on novel chemistry.

Herein, we investigated the reaction of NO with conjugates of electron‐deficient AF drugs and fluorescent dyes (edAF–dye, Figure 1D) in living cells and in vivo. Using the results of this study, we developed modular fluorogenic probes, whose excitation/emission parameters can be tuned based on the application. We demonstrated that these probes can be used for monitoring NO in living cells and for detecting inflammation sites in vivo, where NO levels are typically elevated.

## 2 Results and Discussion

Inspired by previously reported H_2_O_2_‐responsive probes based on AF prodrug–dye conjugates **4a** ^[^ ^17^ ^]^ and **4c**,^[^ ^13^ ^]^ we synthesized edAF–dye conjugates **4b**, **4d**, and **4e**, incorporating three distinct fluorescent dyes: two coumarin derivatives (C1 and C2) and a BODIPY derivative (BDP). Fluorescence of these dyes collectively spans the visible spectrum from 475 to 616 nm. This selection was designed to confirm that the conjugates’ reactivity with NO is independent of the dye. Furthermore, the versatility in dye selection within the edAF–dye conjugates enables their application across various contexts, from monitoring NO in living cells to detecting inflammatory sites in vivo. In contrast to **4a** and **4c**, we replaced the H_2_O_2_‐responsive boronic acid pinacol ester group with a stable dimethylaminocarbonyl (Dma) moiety^[^ ^17^ ^]^ in the edAF–dye conjugates as described in the next section.

### 2.1 Synthesis of edAF–dye Conjugates

**Scheme 1:**
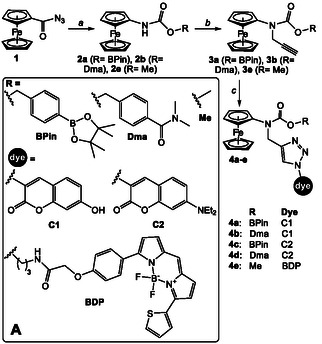
Synthesis of edAF–dye conjugates **4a**–**e**: a) MeOH/toluene, reflux; b) propargyl chloride, Cs_2_CO_3_, CsI; c) CuI / TBTA and either C1–N_3_ (for **4b**) or C2–N_3_ (for **4d**) or BDP‐N_3_ (for **4e**).

Synthesis of conjugates **4a–** **e** is outlined in **Scheme** **1**. First, we prepared carbamates **2a**, **2b,** and **2e** via Curtius rearrangement of ferrocenoylazide **1,** followed by addition of the corresponding alcohols R–OH (step *a*). The resulting intermediates **2** were alkylated with propargyl chloride in the presence of Cs_2_CO_3_ to obtain the corresponding intermediates **3** (step b). Finally, the latter products were coupled to dye‐azides (C1–N_3_, C2–N_3_ or BDP‐N_3_,) under conditions of Cu(I)‐catalyzed alkyne‐azide cycloaddition ‘click’ reaction to obtain conjugates **4a–** **e**, which were chromatographically purified up to >95% purity (supporting information:).

### 2.2 Reaction of edAF–dye's with NO in Cell Free Settings

**Figure 2:**
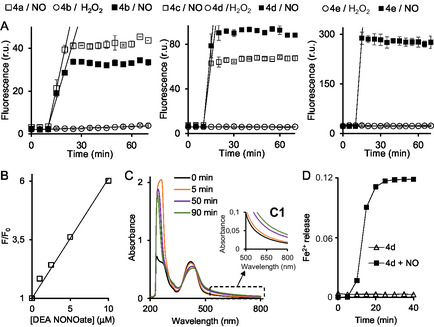
A) Increase of fluorescence intensity of edAF–dye conjugates **4b**, **4d,** and **4e** and controls **4a** and **4c** (each 2.5 μM) in the absence (first 10 min) and the presence (10–90 min) of either an NO donor DEA NONOate (250 μM) or H_2_O_2_ (10 mM). Buffer: PBS / DMSO (995/5, v/v). Initial fluorescence increases (dF/dt) after the addition of the latter reagents are indicated with straight lines, which have the following slopes ( min^−1^): 3.60 (**4a**/NO); 2.63 (**4b**/NO); 0.03 (**4b**/H_2_O_2_); 11.05 (**4c**/NO); 14.41 (**4d**/NO); 0.00 (**4d**/H_2_O_2_); 53.2 (**4e**/NO); −0.01 (**4e**/H_2_O_2_). Parameters of fluorescence detection: derivatives of C1 (**4a**, **4b**) − *λ*_ex_ = 345 nm, *λ*_em_ = 475 nm; derivatives of C2‐dye (**4c**, **4d**) − *λ*_ex_ = 415 nm, *λ*_em_ = 500 nm; the derivative of BDP‐dye (**4e**) − *λ*_ex_ = 589 nm, *λ*_em_ = 616 nm. Three independent experiments (*N* = 3) were conducted to calculate the standard deviations provided in these plots. B) Dependence of fluorescence of **4d** (2.5 μM) from the concentration of NONOate in the range from 0 to 10 μM after 80 min incubation. At [DEA NONOate] > 10 μM, the fluorescence is not increased further. Buffer: PBS / DMSO (995/5, v/v). C) UV–vis spectra of **4d** (25 μM) / DEA NONOate (250 μM) in PBS / DMSO (995/5, v/v) at different incubation times as shown on the plot. D) Release of Fe^2+^ in **4d** (10 μM) / DEA NONOate (250 μM) / ferrozine (100 μM) mixture in PBS (10 mM)/ammonium acetate (100 mM)/MeOH (4/1/4.8, v/v/v) solvent after 40 min incubation at 23.5 ± 1.5 °C. Absorbance at 562 nm was monitored in this experiment.

Similarly to previously known H_2_O_2_‐responsive **4a** and **4c**, fluorescence of three new conjugates **4b**, **4d,** and **4e** in phosphate‐buffered saline (PBS, pH 7.4) is strongly quenched (**Figure** **2**A and S15, Supporting Information). The reason for the quenching is PET from the ferrocenyl moiety to a dye.^[^ ^13^ ^,^ ^17^ ^]^ Thus, if the ferrocenyl moiety is modified in some way, e.g., oxidized or decomposed, the quenching should be lifted. We observed that the fluorescence of **4d** and **4e** is not affected by H_2_O_2_ (black circles in plots shown in Figure 2A). In particular, the same fluorescence is observed in the absence of H_2_O_2_. These data indicate that the ferrocenyl moiety in the latter conjugates is stable toward H_2_O_2_. In contrast, the reaction of **4b** with H_2_O_2_ is more pronounced, though still inefficient (initial fluorescence increase (d*F*/d*t*) = 0.03 min^−1^ vs 0.00 min^−1^ in the absence of H_2_O_2_), confirming limited H_2_O_2_‐mediated oxidation or decomposition of the ferrocenyl moiety. The diethylammonium salt of 1,1‐diethyl‐2‐hydroxy‐3‐oxotriazane (DEA NONOate), a nitric oxide (NO) donor, significantly enhances the fluorescence of all three conjugates—**4b**, **4d**, and **4e** (Figure 2A). When correcting for background activation caused by H_2_O_2_, the fluorescence ratios at 70 min (F_70 min_(+NONOate)/F_70 min_(+H_2_O_2_)) are comparable for conjugates **4b** (8.7) and **4e** (11.7), but are higher for **4d** (14.6).

**Figure 3:**
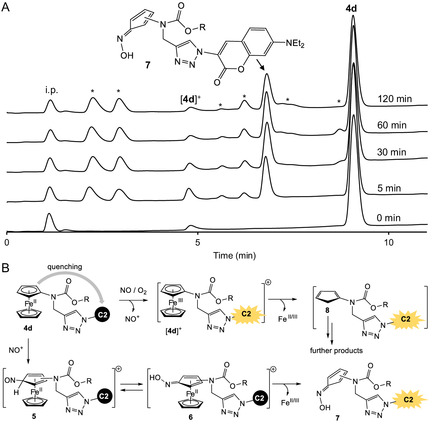
A) Monitoring the reaction of **4d** (50 μM) with the NO donor DEA NONOate (250 μM) by using HPLC (monitoring absorbance at 254 nm). “i.p.”: injection peak. Not identified products are indicated by *. B) Proposed mechanism of the reaction of **4d** with NO under aerobic conditions.

To further investigate NO reactivity, conjugate **4d** was selected for detailed study due to its strong response to NO and no background reaction with H_2_O_2_. The fluorescence of **4d** is increased in a dose‐dependent manner upon exposure to NO (Figure 2B). A linear response was observed up to 10 μM DEA NONOate with a 2.5 μM **4d** solution, beyond which saturation occurred. Under these conditions, the detection limit for DEA NONOate was 1 μM. Next, we elucidated the chemical basis of the NO‐induced fluorescence increase in **4d** by employing three analytical methods: UV–vis spectroscopy, photometric detection of Fe^2+^ ions, and high performace liquid chromatography (HPLC) analysis of reaction products. The UV–vis spectrum of **4d** (25 μM in aqueous solution) is shown in Figure 2C. It displays a UV absorption band at 230 nm, a shoulder near 270 nm, and a broad band at 422 nm—primarily attributed to the coumarin dye C2.^[^ ^13^ ^]^ Although AF absorb in the 410–450 nm range, their extinction coefficients (<100 M^−1^  cm^−1^) are too low to significantly contribute to the spectrum under the given conditions ([**4d**] = 25 μM). 5 min after the addition of DEA NONOate, the UV absorption bands of **4d** exhibited a bathochromic shift and marked intensity increase, consistent with oxidation of the ferrocene moiety to a ferrocenium species.^[^ ^28^ ^]^ In contrast, the 422 nm band slightly decreased in intensity. At incubation times beyond 5 min, the ferrocenium‐related UV band diminished significantly, suggesting decomposition. Concurrently, the baseline increased (Figure 2C1), likely indicating the formation of insoluble, light‐scattering products. Since ferrocenium decomposition is expected to release Fe^2+^ ions as previously described,^[^ ^29^ ^]^ we analyzed a mixture of **4d** and DEA NONOate incubated for 40 min for Fe^2+^ content using photometry in combination with ferrozine as a Fe^2+^‐selective probe (Figure 2D).^[^ ^29^ ^,^ ^30^ ^]^ In the absence of DEA NONOate, conjugate **4d** did not release Fe^2+^ ions, consistent with its overall stability observed in the previously described spectroscopic analysis (Figure 2A). In contrast, in the presence of DEA NONOate, a red color developed in the mixture containing conjugate **4d** and ferrozine, indicating the formation of the Fe^2+^(ferrozine)_3_complex. The kinetics of complex formation were monitored by tracking the increase in absorbance at 562 nm (Figure 2D). We observed that the Fe^2+^ release is completed at around 25 min, which corresponds to the time range when fluorescence of **4d** is most quickly increased (Figure 2A, middle plot). Using HPLC coupled with a UV‐absorbance detector (monitoring absorbance at 254 nm) and offline acquisition of high‐resolution electrospray ionization (HR‐ESI) spectra of the HPLC fractions, we confirmed the formation of the oxidized conjugate [**4d**]^+^ (**Figure** **3**A,B).

We also identified product **7**, which is released as a result of NO‐mediated decomposition of **4d** by detecting [M+H^+^]^+^ ions with *m/z* 612.2563 (calculated for C_32_H_34_N_7_O_6_ 612.2565) and [M+Na^+^]^+^ ions with *m/z* 634.2388 (calculated for C_32_H_33_N_7_O_6_Na 634.2385) (Figure 3A and S1–S3, Supporting Information). The proposed mechanism of product **7** formation is outlined in Figure 3B. It involves the reaction of electrophilic NO^+^, derived from the reaction of NO with air oxygen, with the ferrocenyl fragment of **4d**, leading to the formation of the nitroso intermediate **5**, which exists in equilibrium with its oxime tautomer **6**. The modified Cp ligands in both **5** and **6** are neutral and are expected to coordinate Fe^2+^ with substantially lower affinity than the original Cp^−^ ligands. This reduced binding affinity promotes complex decomposition, resulting in the release of free Fe^2+^ ions and ligand‐derived oxime **7**, along with other products derived from Cp^−^. Additionally, to Fe^2+^ (Figure 2D) and product **7** (Figure 3A), we observed four unidentified products by HPLC, which are likely Cp^−^‐derived degradation products. Collectively, these findings support the proposed mechanism.

It is known that ferrocene can be oxidized by NO/O_2_mixtures to form ferrocenium ions.^[^ ^21^ ^]^ This reaction likely accounts for the formation of [**4d**]^+^ in the mixture of DEA NONOate and **4d** (Figure 3A). Ferrocenium [**4d**]^+^ is a 17‐electron complex that is inherently unstable and decomposes to yield free Fe^3+^ ions and Cp ligands. The liberated Cp ligands are susceptible to air oxidation and may undergo cycloadditions with each other, as previously reported for related ferrocene derivatives.^[^ ^28^ ^]^ However, we were unable to identify such products, likely due to their poor ionizability under the mass spectrometric conditions employed.

**Figure 4:**
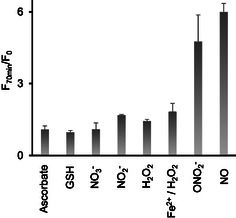
Evaluation of the selectivity of **4d** towards NO. Fluorescence (*λ*_ex_ = 415 nm, *λ*_em_ = 500 nm) was detected before (F_0_) and after the addition of the specific reagent to **4d** and incubation for 70 min (F_70 min_). Reagent concentrations—sodium ascorbate (indicated “ascorbate”): 1 mM, glutathione (“GSH”)‐10 mM, KNO_3_‐100 μM, KNO_2_‐100 μM, H_2_O_2_‐1 mM, H_2_O_2_ (1 mM) followed by addition of FeSO_4_ (100 μM) (to generate HO·), Sin1‐10 μM) (to generate ONO_2_^−^), and DEA NONOate‐10 μM (to generate NO).

Next, we investigated whether the fluorescence increase in solutions of **4d** is specific for NO. We compared the effects of an NO with those of compounds present in living cells at their typical intracellular concentrations. H_2_O_2_ and ONOO^−^ (derived from 3‐morpholinylsydnonimine chloride, Sin1), which are main competitors of NO in cells, were used in >10‐fold higher concentrations. In particular, we tested the effects of ascorbate (1 mM), glutathione (GSH, 10 mM), NO_3_^−^ (100 μM), NO_2_^−^ (100 μM), H_2_O_2_ (1 mM), HO· (generated by mixing H_2_O_2_, 1 mM, and Fe^2+^, 100 μM), ONO_2_^−^ (generated from Sin1, 10 μM), and NO (generated from DEA NONOate, 10 μM). The data obtained are shown in **Figure** **4**. We found that **4d** exhibits no statistically significant increase of fluorescence in the presence of all tested reagents except for NO and ONO_2_^−^ (Student's t test, *p* < 0.001, reference: **4d** without any additives). Effects of NO and ONO_2_^−^ were identical (Student's t test, *p* > 0.05). Thus, **4d** is selective toward the reactive nitrogen species (RNS) rather than NO alone.

### 2.3 Monitoring NO in Cells Using Conjugates 4d and 4e and in Vivo ‐ Using 4e

**Figure 5:**
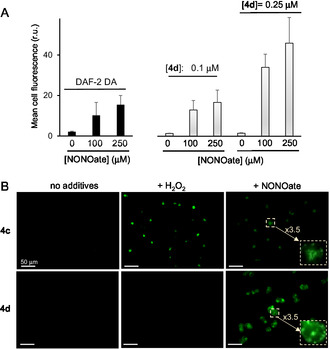
A) Fluorescence of A2780 cells loaded either with 4,5‐diaminofluorescein diacetate (DAF‐2 DA) or **4d** and treated with DEA NONOate: *λ*_ex_ = 405 nm, *λ*_em_ = 485–565 nm, determined by flow cytometry. B) Monitoring fluorescence of A2780 cells loaded either with **4c** or **4d** and treated with DEA NONOate (250 μM) by using fluorescence microscopy: *λ*_ex_ = 470 ± 20 nm, *λ*_em_ = 525 ± 25 nm.

Encouraged by the promising data obtained in cell‐free settings for compound **4d**, we proceeded to investigate whether this conjugate responds to elevated intracellular NO levels. For this purpose, we selected a representative cancer cell line—human ovarian carcinoma A2780 cells—which are known to produce high levels of ROS.^[^ ^8^ ^,^ ^9^ ^]^ This makes them a challenging model for NO probes, which must effectively distinguish NO signals in the presence of a high ROS background. To elevate NO levels in A2780 cells, we treated them with the NO donor DEA NONOate (100–250 μM). We confirmed that this treatment leads to an increase in intracellular NO using the established NO probe DAF‐2 DA (**Figure** **5**A).

We were pleased to observe that fluorescence intensity in A2780 cells preloaded with either 0.1 or 0.25 μM of **4d** significantly increased following treatment with the NO donor (Student's t test, *p* < 0.001), as determined by flow cytometry (Figure 5A). This result was further corroborated by fluorescence microscopy (Figure 5B). Interestingly, the previously reported H_2_O_2_‐responsive probe **4c**, which relies on the reactivity of arylboronic pinacol ester with H_2_O_2_, also showed a response to the DEA NONOate‐induced increase in NO. In contrast, probe **4d** displayed specificity to NO and did not respond to H_2_O_2_ treatment (Figure 4B). Importantly, we observed that probe **4d** is not cytotoxic toward A2780 cells up to the concentration of 25 μM (Supporting Information).

**Figure 6:**
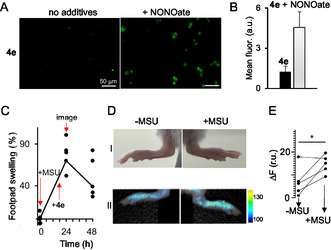
A) Fluorescence of A2780 cells loaded with **4e** (0.5 μM) and either directly imaged (“no additives”) or first treated with DEA NONOate and then imaged (“+NONOate). Imaging parameters: *λ*_ex_ = 640 ± 15 nm, *λ*_em_ = 690 ± 25 nm. B) Quantification of fluorescent signals in images shown in **A**. The difference between groups “**4e**” and “**4e**+NONOate” is statistically significant: Student's t test, *p* < 0.05. C) Swelling of the footpads of mice paws, which were injected with MSU. The plot outlines the design of the in vivo experiment: times of injection of MSU, conjugate **4e,** and the time of imaging are indicated with red‐colored arrows. D) I‐Representative photos of footpads of mice paws taken 24 h after MSU (“+MSU”) or NaCl (“‐MSU”) injection. II‐Fluorescence of the corresponding footpads, whose photos are provided in D‐I. E) Quantification of fluorescence of footpads of mice paws (*N* = 5). Representative images are shown in D‐II. *: Student t test, *p* < 0.05.

Additionally, we tested conjugate **4e** as a probe for intracellular NO in the above model. We observed that this conjugate responds to intracellular NO in A2780 cells (**Figure** **6**A,B). Since products of the reaction of NO with **4e** emit >600 nm, this probe could be potentially useful for the detection of NO and NO‐derived product ONO_2_^−^ in vivo. To test whether this is the case, we employed the murine model of acute inflammation mimicking gouty arthritis as previously described.^[^ ^31^ ^]^ In this model, one of the paws of a the wild‐type mouse is injected with a suspension of monosodium urate (MSU) micro‐needles to induce neutrophil/neutrophil‐extracellular‐trap driven inflammation (rich in ROS/RNS) in the site of injection, and visualized in vivo to contain abundant neutrophil elastase activation,^[^ ^29^ ^]^ that leads to paw swelling, which persists at least 48 h (Figure 6C,D). Another paw is injected with saline and used as a reference control.

Conjugate **4e** is injected 18 h after the inflammation induction by MSU, and both paws (inflamed and control) are imaged by using LiCor Pearl Trilogy imager instrument (fluorescence parameters using excitation at 685 nm and emission at 720/20 nm) (Figure 6D,E). We analyzed the effects in five mice by using this protocol. We found that the fluorescence signal is statistically significantly (Student's t test, *p* < 0.05) increased in MSU‐treated paws as compared to PBS‐treated paws. These data confirm the applicability of **4e** for monitoring inflammation in vivo. Systemic availability of the compounds leads to a response that can be monitored locally (only in the inflamed paw). At the same time, the compatibility of **4e** with the optical setup used for Cy5 and Cy5.5 imaging makes it a useful option for in vivo localization of RNS.

The study received approval from the Ethics Committees of ICBP‐NS (approval no. 3/June 06, 2022), the Romanian National Sanitary Veterinary and Food Safety Authority (authorization no. 31/September 12, 2022), and DH‐LNMU (Authorization no 9/ December 21, 2020).

## 3 Conclusion

We synthesized three novel conjugates of edAF drugs with fluorescent dyes: two coumarins (C1 and C2) and one BDP. In their native state, the fluorescence of these conjugates is strongly quenched due to PET from the ferrocenyl moiety to the attached dye. Upon exposure to nitric oxide (NO), this quenching is reversed, and all three conjugates become strongly fluorescent, with emission wavelengths ranging from 475 to 616 nm, depending on the dye. For the selected conjugate **4d**, we demonstrated that: 1) NO induces a dose‐dependent increase in fluorescence; 2) **4d** is sensitive to NO concentrations as low as 1 µM; 3) in addition to NO, **4d** also responds to peroxynitrite, but shows no significant response to a range of other endogenous species, including ascorbate, glutathione, nitrate, nitrite, hydrogen peroxide, and hydroxyl radicals; and 4) the reaction between **4d** and NO leads to its oxidation and subsequent decomposition, yielding oxime **7** and iron ions as products. Although the interaction of ferrocene with NO has been studied previously, prior investigations were conducted under nonphysiological conditions (e.g., pH ≈3), where only ferrocenium (Fc^+^) and the product Fc^+^—ON = NO—Fc^+^ were observed. In contrast, we report the formation of oxime **7** for the first time, providing new insight into the potential intracellular metabolism of edAFs. We propose that similar NO‐mediated transformations may occur with other ferrocene‐containing drugs and prodrugs, including those currently in preclinical (e.g., HFce derivatives and analogs) and clinical (e.g., FQ) development, as well as other experimental ferrocene‐based therapeutics. This possibility remains to be confirmed experimentally for the latter compounds. Finally, leveraging this NO‐triggered reactivity, we developed functional fluorescent probes for detecting intracellular NO (probe **4d**) and for monitoring inflammation sites in vivo (probe **4e**).^[32,33]^

## Supporting Information

Synthetic protocols, characterization of new compounds, descriptions of assays in cell free settings, in cells and in vivo are included to the SI. The authors have cited additional references within the SI.^[32,33]^

## Conflict of Interest

The authors declare no conflict of interest.

## Supporting information

Supplementary Material

## Data Availability

The data that support the findings of this study are available in the supplementary material of this article.
