# Supplementary material for: Reaction of N‐Ferrocenylcarbamates with Nitric Oxide: An Application for Detection of Inflammatory Sites In Vivo
Source: ChemMedChem. 2025 Aug 22;20(19):e202500356. doi: 10.1002/cmdc.202500356 (PMC12503903; doi:10.1002/cmdc.202500356)
Supplement: Supplementary file 1 — Supplementary Material [file CMDC-20-e202500356-s001.pdf]

SUPPORTING INFORMATION

Reaction of N-Ferrocenylcarbamates with Nitric Oxide: An Application for Detection of Inflammatory Sites In Vivo

Roman Selin, Hülya Gizem Özkan, Galyna Bila, Rostyslav Bilyy, Andriy Mokhir*

Content

General	S2
Synthesis	S2-S5
Studies of edAF-dye conjugates in cell free settings	S5-S8
In vitro assays	S9-S10
In vivo experiments	S10
Characterization data for new conjugates	S11-S16

General

Commercially available chemicals of the best quality from Sigma-Aldrich (Germany) Alfa-Aesar (Germany) and Lumiprobe (Germany) were obtained and used without purification. NMR spectra were acquired on a Bruker Avance 300 or Bruker Avance 400 spectrometers (Ettlingen, Germany). ESI mass spectra were recorded on a Bruker ESI MicroTOF II (Bremen, Germany) or Bruker maXis 4G mass spectrometers (Bremen, Germany). C/H/N elemental analysis was performed in the microanalytical laboratory of the Department Chemistry and Pharmacy, Organic Chemistry Chairs 1 and 2 of the Friedrich-Alexander-University of Erlangen-Nürnberg. UV-visible spectra were measured on a Cary 100 UV-visible spectrophotometer (Agilent Technologies, Frankfurt am Main, Germany) by using either quartz glass cuvettes (Hellma GmbH, Müllheim, Germany) with a sample volume of 1 mL or micro-cuvettes with a sample volume of 100 µL (BRAND GmbH, Wertheim, Germany). Fluorescence spectra were acquired on a Varian Cary Eclipse fluorescence spectrophotometer using fluorescence cuvettes (Hellma GmbH, Müllheim, Germany) with a sample volume of 1 mL. The fluorescence images were taken with a Zeiss Axio Vert.A1 with 40x/1.30 oil objective (DIC), and the following filter set: Channel 1 (Ch1, UV) λ_{ex}= 335–383; λ_{em}= 420–470 nm, Channel 2 (Ch2, Blue) λ_{ex}= 450–490; λ_{em}= 500–550 nm, Channel 3 (Ch3, Green) λ_{ex}= 538–562; λ_{em}= 570–640 nm, and Channel 4 (Ch4, Red) λ_{ex}= 625–655; λ_{em}= 665–715 nm.

Synthesis

3-azido-7-hydroxycoumarin, 3-azido-7-diethylaminocoumarin,[32] intermediate **3b**[17] and edAF~dye conjugates **4a**[17] and **4c**[13] were synthesized according to previously described procedures.

*Conjugate **4b***

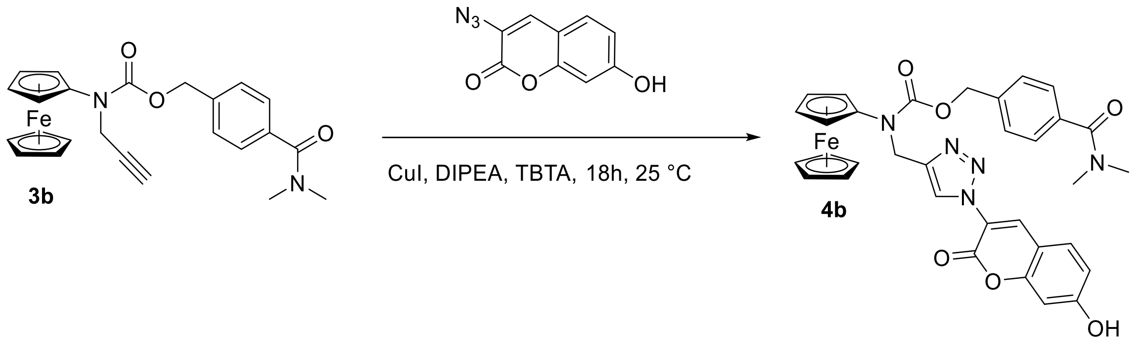

In 250 mL round bottom flask, intermediate **3b** (210 mg, 472.64 µmol) was dissolved in anhydrous dichloromethane (3 mL) and anhydrous acetonitrile (20 mL) under N_2 atmosphere. DIPEA (411.64 µL, 2.36 mmol) and 3-azido-7-hydroxycoumarin (96.02 mg, 472.64 µmol) were added. In another vial, CuI (9 mg, 47.26 µmol) and TBTA (25.08 mg, 47.26 µmol) were dissolved in anhydrous acetonitrile (4 mL) and added into the reaction mixture. The mixture was stirred 18h at 25°C, the solvent was removed and crude product purified via column chromatography (DCM:MeOH, 90:10, v/v). The product was obtained as orange-brownish solid: 220 mg, 340 µmol, 72%). ^{1}H NMR spectroscopy (500 MHz, solvent: DMSO-*d*$_6$): δ 10.88 (s, 1H), 8.59 (s, 1H), 8.44 (s, 1H), 7.75 (d, *J* = 8.6 Hz, 1H), 7.48 – 7.33 (m, 4H), 6.90 (dd, *J*

= 8.6, 2.2 Hz, 1H), 6.85 (d, *J* = 2.2 Hz, 1H), 5.21 (s, 2H), 5.04 (s, 2H), 4.59 (s, 2H), 4.12 (s, 5H), 4.06 – 3.98 (m, 2H), 2.94 (s, 3H), 2.86 (s, 3H). ^{13}C NMR (101 MHz, DMSO-d_6) δ 169.73, 162.37, 156.28, 154.62, 144.36, 137.52, 136.16, 136.02, 130.94, 127.57, 127.09, 124.15, 119.33, 114.22, 110.34, 102.12, 68.79, 66.59, 64.24, 62.37, 54.89, 34.69. HR-MS (ESI+), *m/z:* calculated for $C_{33}H_{29}FeN_5O_6$ ([M-e$^-$]$^+$) 647.1467, found 647.1453. C, H, N analysis: calculated for $C_{33}H_{29}FeN_5O_6$ (%) – C 61.22, H 4.51, N 10.82, found – C 60.79, H 4.76, N 10.44.

*Conjugate **4d***

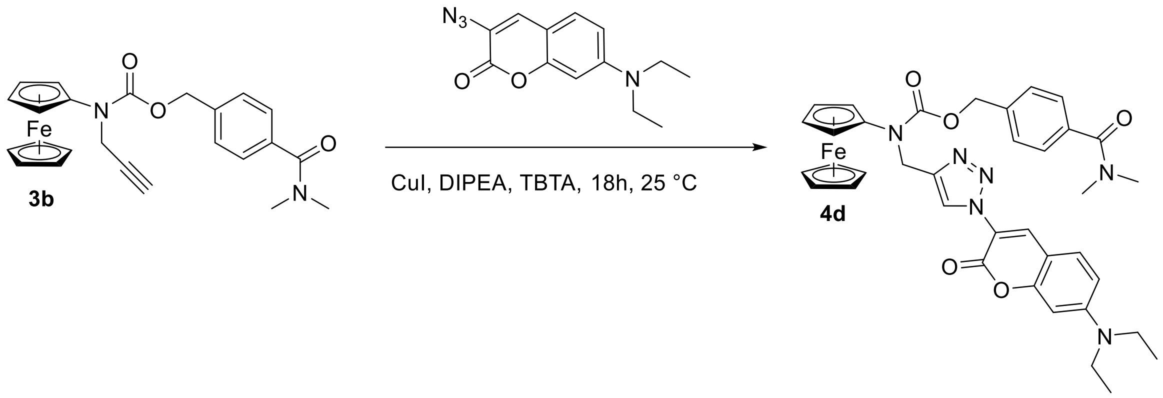

In 250 mL round bottom flask, intermediate **3b** (250 mg, 562.67 µmol) was dissolved in anhydrous dichloromethane (3.2mL) and anhydrous acetonitrile (25 mL) under N_2 atmosphere. DIPEA (490 µL, 2.81 mmol) and 3-azido-7-diethylaminocoumarin (145 mg, 562.67 µmol) were added. In another vial, CuI (10.72 mg, 56.27 µmol) and TBTA (29.86 mg, 56.27 µmol) were dissolved in anhydrous acetonitrile (4 mL) and added into the reaction mixture. After it was stirred 18h at 25°C, the solvent was removed and crude product was purified via column chromatography (DCM:MeOH, 90:10, v/v). The product was obtained as orange-brownish solid: 313 mg, 445.49 µmol, 79%. ^{1}H NMR spectroscopy (400 MHz, DMSO-d_6) δ 8.46 (s, 1H), 8.40 (s, 1H), 7.63 (d, *J* = 9.0 Hz, 1H), 7.46 – 7.36 (m, 4H), 6.82 (dd, *J* = 9.0, 2.4 Hz, 1H), 6.67 (d, *J* = 2.4 Hz, 1H), 5.23 (s, 2H), 5.04 (s, 2H), 4.60 (s, 2H), 4.13 (s, 5H), 4.04 (t, *J* = 2.0 Hz, 2H), 3.47 (q, *J* = 7.0 Hz, 4H), 2.90 (d, *J* = 34.4 Hz, 6H), 1.14 (s, 6H). ^{13}C NMR spectroscopy (101 MHz, DMSO-d_6) δ 169.73, 156.69, 155.63, 151.43, 144.20, 137.54, 136.65, 136.02, 130.53, 127.58, 127.09, 124.05, 116.18, 109.99, 106.46, 96.36, 68.80, 68.80, 66.59, 64.24, 62.35, 54.90, 44.20, 34.70, 12.29. HR-MS (ESI+), *m/z:* calculated for $C_{37}H_{38}FeN_6O_5$ ([M-e$^-$]$^+$) 702.2248, found 702.2253. C, H, N analysis: calculated for $C_{37}H_{38}FeN_6O_5$ x 0.5 H_2O (%) – C 62.45, H 5.52, N 11.81, found – C 62.60, H 5.46, N 11.73.

Intermediate ***2e***

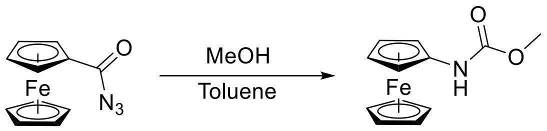

1 **2e**

Ferrocenoyl azide **1** (200 mg, 784.1 µmol) was dissolved in MeOH/toluene (1:1, v:v) mixture (5 mL). The reaction mixture were refluxed for 3 hours. Solvents were evaporated under vacuum and crude product was purified via column chromatography (DMC:Cyclohexane from 1:1 till 1:0, v:v). The product **2e** was obtained as yellow solid: 140 mg, 540.4 µmol, 69%. ^{1}H NMR spectroscopy (400 MHz, DMSO-*d$_6$*): δ 8.81 (s, 1H), 4.46 (s, 2H), 4.11 (s, 5H), 3.93 (t, J = 1.9 Hz, 2H), 3.61 (s, 3H). ^{13}C NMR spectroscopy (101 MHz, DMSO-*d$_6$*): δ 154.68, 97.22, 69.17, 64.01, 60.35, 51.97. HR-MS (ESI+), *m/z:* calculated for $C_{12}H_{13}FeNO_2$ ([M-e$^-$]$^+$) 259.0296, found: 259.0304.

Intermediate ***3e***

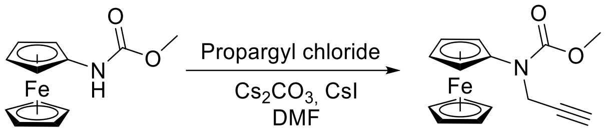

2e **3e**

Intermediate **2e** (200 mg, 0.772 mM) was dissolved in dry DMF (2 mL) under argon stream. Cesium carbonate (503 mg, 1.544 mM, 2 eq), cesium iodide (200.6 mg, 0.772 mM, 1 eq) and propargyl chloride (6.7 µL, 0.926 mM, 1.2 eq) were added reaction mixture and it was stirred at 55° C overnight. Solvent was removed under vacuum and reaction mixture was purified via column chromatography (DCM:MeOH from 1:0 till 95:5, v:v). The product **3e** was obtained as yellow solid: 165 mg, 555.3 µmol, 72%. ^{1}H NMR (400 MHz, DMSO-*d$_6$*) δ 4.52 (s, 2H), 4.44 (d, J = 2.5 Hz, 2H), 4.23 (s, 5H), 4.07 (t, J = 2.0 Hz, 2H), 3.69 (s, 3H), 3.32 (t, J = 2.7 Hz, 1H). ^{13}C NMR (101 MHz, DMSO-*d$_6$*) δ 81.16, 75.04, 69.37, 64.77, 53.46. HR-MS (ESI+), *m/z:* calculated for $C_{15}H_{15}FeNO_2$ ([M-e$^-$]$^+$) 297.0452, found: 297.0438.

*Conjugate **4e***

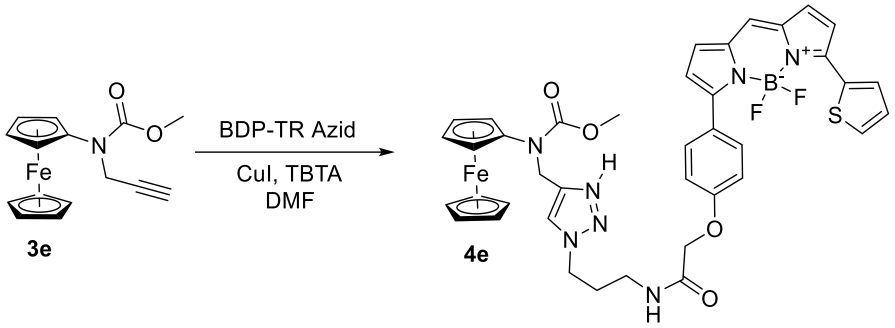

Solutions of **3e** (100 µL, 1 µmol, 1 eq.), BDP-N_3 (Scheme 1, main text) (100 µL, 1 µmol, 1 eq.) and CuI/TBTA complex (25 µL, 0.25 µmol, 0.25 eq.), each 0.01 M in dry DMF, were mixed together under argon atmosphere in the test tube, sealed and covered with aluminum foil to protect it from UV-light. The reaction mixture was shaken at 1100 rpm at 55 °C overnight. DMF was evaporated under reduced pressure from the test tube and the mixture was purified using HPLC. Conjugate **4e** was obtained as blue solid: 0.456 mg, 550 nmol, 55%. ^{1}H NMR (601 MHz, Methylene Chloride-d_2) δ 8.04 (dd, *J* = 3.8, 1.1 Hz, 1H), 8.00 – 7.95 (m, 2H), 7.61 (s, 1H), 7.53 (dd, *J* = 5.1, 1.0 Hz, 1H), 7.26 (s, 1H), 7.18 – 7.14 (m, 2H), 7.14 – 7.11 (m, 1H), 7.11 – 7.06 (m, 2H), 6.85 (dt, *J* = 4.4, 0.8 Hz, 1H), 6.82 (s, 1H), 6.72 – 6.68 (m, 1H), 4.97 (s, 2H), 4.59 (s, 2H), 4.56 (s, 2H), 4.36 (t, *J* = 6.8 Hz, 2H), 4.14 (s, 5H), 3.99 (t, *J* = 2.0 Hz, 2H), 3.74 (s, 3H), 3.38 (q, *J* = 6.5 Hz, 2H), 2.15 (p, *J* = 6.7 Hz, 2H). ^{13}C NMR (151 MHz, CD_2Cl_2) δ 167.89, 158.46, 158.04, 158.03, 157.78, 150.66, 150.64, 145.31, 136.77, 133.96, 131.38, 131.24, 130.21, 130.08, 129.65, 128.82, 126.42, 126.38, 120.76, 114.53, 68.91, 67.33, 64.48, 52.75, 47.68, 36.06, 30.17. HR-MS (ESI+) *m/z* calculated for $C_{39}H_{37}BF_2FeN_7O_4S$ ($[M+H^+]^+$) 804.2032, found: 804.2027. Analytical HPLC (method A): retention time (R_t)= 8.87 min.

Studies of edAF-dye conjugates in cell free settings

Chemical transformation of edAF-dye's and controls in the presence of NO donor DEA NONOate.

Monitoring fluorescence increase of edAF-dye conjugates and controls in the presence of NO and H_2O_2

Stock solutions of conjugates **4a**, **4b**, **4c** or **4d** in DMSO (500 µM, 5 µL) were added to PBS (995 µL, pH 7.4) in a quartz cuvette and thoroughly mixed. Fluorescence of the resulting solutions was detected every 5 min. After 10 min, either DEA NONOate (25 mM, 10 µL) or H_2O_2 (1 M, 10 µL) solutions in water was added and the measurement was continued for 80 min (left and middle plots in Figure 2A, main text). Final concentrations of the conjugates were 2.5 µM, DEA NONOate – 250 µM and H_2O_2 – 10 mM. The experiment with conjugate **4e** was conducted similarly, except that it was used at the lower final concentration of 0.25 µM (right plot in Figure 2A, main text). Parameters of fluorescence detection: for derivatives of C1-dye (**4a**, **4b**) - λ_{ex}= 345 nm, λ_{em}= 475 nm; for derivatives of C2-dye (**4c**, **4d**) - λ_{ex}= 415 nm, λ_{em}= 500 nm; for the derivative of BDP-dye (**4e**) - λ_{ex}= 589 nm, λ_{em}= 616 nm. All measurements and incubations were conducted at 22 °C.

Dependence of the fluorescence increase of conjugate **4d** from concentration of NO in solution

Stock solution of **4d** in DMSO (500 µM, 5 µL) was added to PBS (995 µL, pH 7.4) in a quartz cuvette and thoroughly mixed. Next, DEA NONOate solutions in water (10 µL) at different concentrations (0.1 or 0.25 or 0.5 or 1 mM) were added. After incubation for 80 min at 22 °C, fluorescence (λ_{ex}= 415 nm, λ_{em}= 500 nm) was detected and plotted as a function of DEA NONOate concentration (Figure 2B, main text).

Monitoring reaction of **4d** with NO by UV-visible spectroscopy

Stock solution of **4d** in DMSO (5 mM, 5 µL) was added to PBS (995 µL, pH 7.4) in a quartz cuvette and thoroughly mixed. UV-visible spectrum of this solution was acquired to obtain kinetic time point 0. Next, DEA NONOate solution in water (25 mM, 10 µL) was added and UV-visible spectra of the resulting solution were acquired after 5, 50 and 90 min incubation at 22 °C (Figure 2C, main text).

Monitoring release of Fe^{2+} in the mixture of **4d** with NONOate

Either stock solution of **4d** in MeOH (1 mM, 10 µL) or MeOH (10 µL) was added to the mixture of PBS (10 mM, 400 µL, pH 7.4), MeOH (480 µL) and ferrozine solution in ammonium acetate buffer (100 mM) (10 mM, 100 µL). After thorough mixing, absorbance of light at 562 nm (A_{562nm}) by this mixture was measured at 23.5 ± 1.5 ^{o}C. Following 10 min incubation, stock solution of DEA NONOate in PBS (25 mM, 10 µL) was added and measurement of A_{562nm} was continued for over 40 min. A_{562nm} correlates with the release of Fe^{2+} ions (Figure 2D, main text).

Identification of products in the reaction of **4d** with DEA NONOate using HPLC-UV-MS

Stock solution of conjugate **4d** in DMSO (5 mM, 5 µL) was added to the mixture of CH_3CN (115 µL) and triethylammonium acetate buffer (100 mM, pH= 7.4, 375 µL). Then, either H_2O (control) or stock solution of DEA NONOate in water (25 mM, 5 µL) was added. The mixture obtained was incubated for 0, 5, 30, 60 and 120 min at 22 °C and analyzed by using HPLC coupled to UV and MS detectors: absorbance at 254 nm *versus* elution time is shown in Figure 3A, main text).

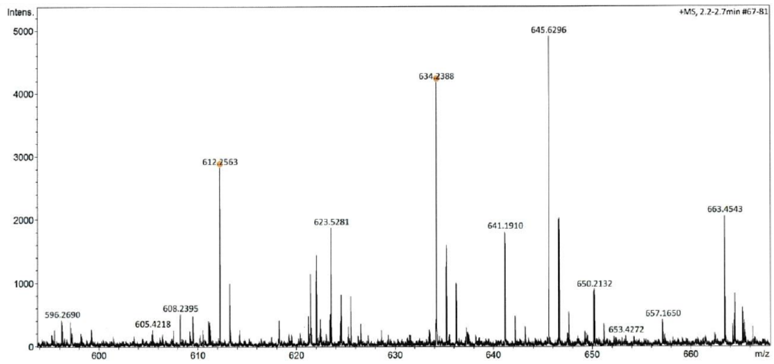

Figure S1. High resolution APPI mass spectrum of fractions between 6.5 and 7.5 minutes (incubation time 120 min). The corresponding HPLC profile is shown in Figure 3A (main text).

A:

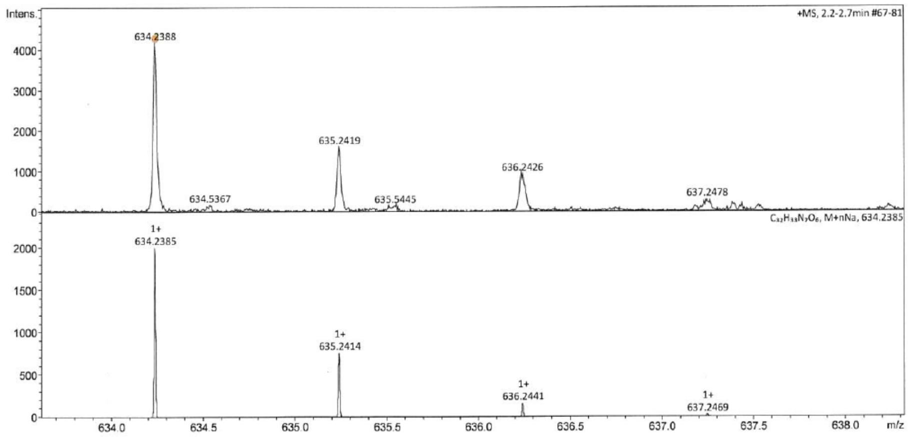

B:

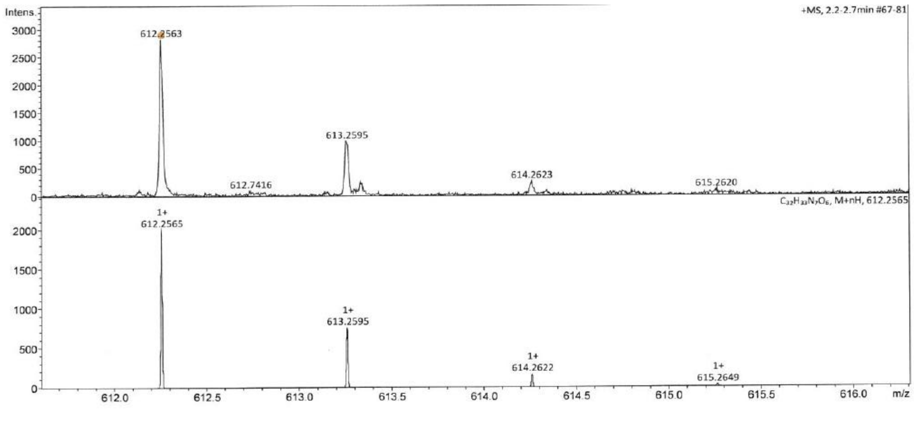

Figure S2. A: Upper plot: a zoomed-in area of high resolution APPI mass spectrum shown in Figure S1. Bottom plot – theoretical spectrum of ion $C_{32}H_{33}N_7O_6Na$ $[M+Na^+]^+$ (see Figure S3 for its structure).
B: Upper plot: a zoomed-in area of high resolution APPI mass spectrum shown in Figure S1. Bottom plot – theoretical spectrum of ion $C_{32}H_{34}N_7O_6$ $[M+H^+]^+$ (see Figure S3 for its structure).

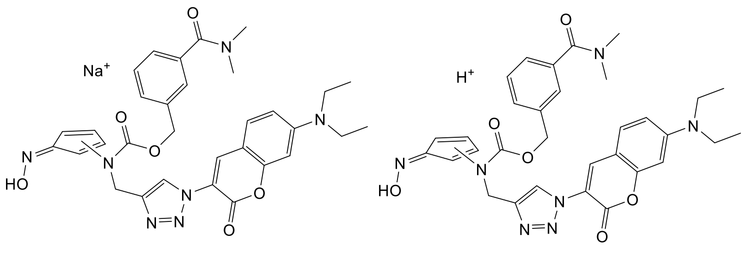

Figure S3. Structures of ions detected in mixtures of **4d** and DEA NONOate by mass spectrometry (Figures S1, S2).

The mixtures obtained in the reaction of NO with conjugate **4e** were analyzed similarly (Figure S4).

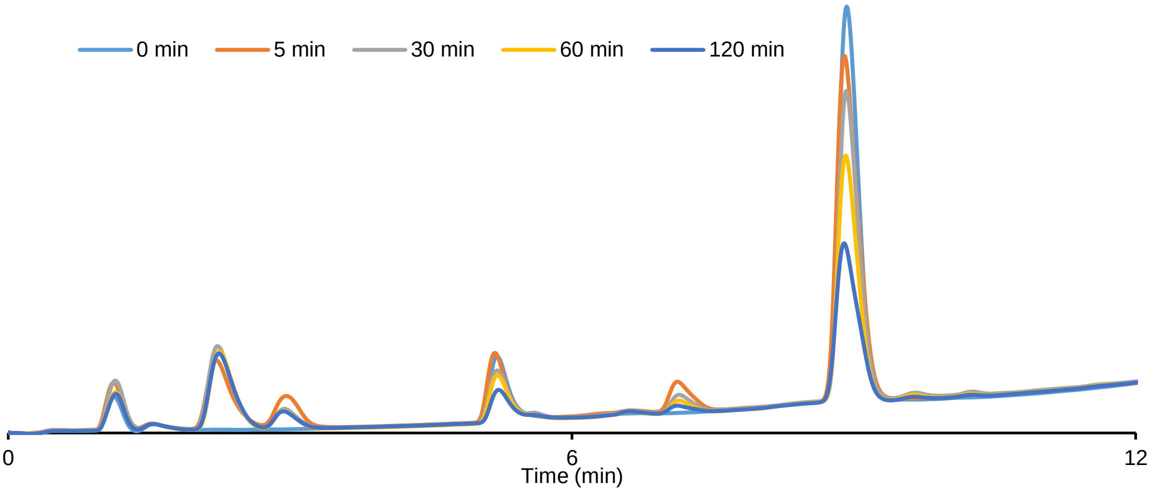

Figure S4. HPLC (monitoring absorbance at 254 nm) of mixtures of **4e** (50 μM) and DEA NONOate (250 μM) after 0, 5, 30, 60 and 120 min incubation at 22 °C. The fraction eluted at 7.2 min corresponds to the oxime derived from **4e**, which is detected as ions with *m/z* 713.00 ($[M+H^+]^+$) and 735.00 ($[M+Na^+]^+$). Structures of an analogous oximes derived from **4d** are shown in Figure S3.

Study of selectivity of **4d** towards NO (Figure 4, main text)

Stock solution of **4d** in DMSO (500 μM, 5 μL) was added to PBS (995 μL, pH 7.4) in a quartz cuvette, thoroughly mixed and fluorescence (λ_{ex}= 415 nm, λ_{em}= 500 nm) was measured (F_0). Next, different reagents were added. Final concentration of sodium ascorbate (indicated "ascorbate") was 1 mM, glutathione ("GSH") – 10 mM, KNO_3 – 100 μM, KNO_2 – 100 μM, H_2O_2 – 1 mM, a mixture of H_2O_2 (1 mM) and $FeSO_4$ (100 μM) (to generate HO·), Sin1 – 10 μM) (to generate ONO_2^-) and DEA NONOate – 10 μM (to generate NO). After incubation for 70 min at 22 °C, fluorescence (λ_{ex}= 415 nm, λ_{em}= 500 nm, F_{70min}) was detected and ratio F_{70min}/F_0 was plotted as a function of the reagent used in the assay.

In vitro assays

Cells and cell culture

Human ovarian cancer cell line A2780 was purchased from Sigma-Aldrich. A2780 cells were grown in RPMI 1640 medium supplemented with 10% FBS, 1% L-glutamine, and 1% penicillin/streptomycin. A2780 was cultivated to 80−90% confluence and detached from the flask by using trypsin/N,N,N',N'-ethylenediaminetetracetic acid (EDTA) solution (0.025% / 0.01%, w/v) in PBS.

Monitoring transformation of **4d** and reference 4,5-diaminofluorescein diacetate in A2780 cells by using flow cytometry

After the cultivation, A2780 cells were resuspended in the RPMI 1640 medium containing 5% FBS, 1% L-glutamine and 1% penicillin/streptomycin. This suspension (250 cells/µL, 100 µL/well) was pipetted one day before the experiment in a 96-well microtiter plate and left at 37 °C in the chamber filled with CO_2 (5%) overnight for the attachment. The cells were incubated for 1h either with **4d** (100 or 250 nM) or 4,5-diaminofluorescein diacetate (DAF-2 DA, 10 nM), washed and treated with DEA NONOate (0, 100 or 250 µM) for 30 min. The cells were then washed, trypsinated and resuspended in fresh medium (100 µL/well). Fluorescence intensity (λ_{ex}= 405 nm; λ_{em}= 485-565 nm) was measured by using CytoFLEX, Beckmann Coulter. The data were analyzed using CytExpert software.

Monitoring transformation of **4c**, **4d** and **4e** in A2780 cells by using fluorescence microscopy

A2780 cells were seeded on a 35 mm imaging dish (µ-Dish 35 mm, high, ibidi GmbH, Germany) at a cell density of 80 cells/µL one day before the experiment in RPMI 1640 medium containing 5% FBS, 1% L-glutamine and 1% penicillin/streptomycin (500 µL). On the next day, the medium was replaced with a fresh portion of RPMI 1640 medium (2 mL). Conjugate **4c** (end concentration: 2.5 µM) or **4d** (end concentration: 5 µM) or **4e** (end concentration: 0.5 µM) in DMSO were added and incubated at 37 °C in the incubator containing 5% CO_2 for 1 h. Afterwards, the cells were washed and DEA-NONOate (end concentration 250 µM) was added and the cells further incubated for 30 min. Finally, cells were washed with DPBS (2 x 2 mL) and the fresh medium (2 mL) was added. The fluorescence images were taken with a Zeiss Axio Vert.A1 and the following filter sets: for **4c** and **4d** - excitation / emission 450-490 / 500-550 nm; for **4e** - excitation / emission 625–655 / 665–715 nm. Objective: 40x/1.30. Oil DIC.

Determination of viability of A2780 in the presence of **4d** via MTT assay

A2780 was grown as mentioned before. Then, they were resuspended in the RPMI medium containing 5% FBS. This suspension was pipetted as 100 µL/well in a 96-well microtiter plate as 25000 cells/100µL. A2780 cells were seeded one day before for the attachment and kept at 37 °C in the chamber filled with CO_2 (5%) overnight. Next, the stock solutions of **4d** dissolved in DMSO at 5 mM, 2.5 mM, 1 mM, 0.5 mM and 0.1 mM were added to the wells giving a final concentration of 50 µM, 25 µM, 10 µM, 5 µM and 1 µM and incubated for 48h. Afterwards, 20 µL/well 3-(4,5-dimethylthiazol-2-yl)-2,5-diphenyltetrazolium bromide (MTT) solution (5 mg/mL in DPBS) was added. After another 3 h incubation under the same

conditions, the cells were treated with 90 µL/well sodium dodecyl sulfate (SDS, 10% solution in 0.01 M aqueous HCl) and incubated overnight. The next day, the intensity of absorbance at 590 nm was measured and the absorbance at 690 nm was taken as a baseline value in the plate reader. These data were processed to calculate the relative number of viable cells. We observed that **4d** is not toxic under the selected conditions up to the concentration of 25 µM (97 $\pm$ 9 % cells remained viable: the cells treated with the carrier were used as a reference: 100 %). At 50 µM of **4d** the number of viable cells was 90 $\pm$ 11 %.

In vivo experiments

All animal experiments were designed to comply with principles of the 3Rs (Replacement, Reduction, and Refinement). Mice were housed in a temperature / humidity / light-controlled environment, with both food and drinking water available ad libitum. The animal studies were approved by the local ethical committees and conducted according to the guidelines of the Federation of European Laboratory Animal Science Associations (FELASA).

Monosodium urate (MSU) crystals initiate abundant NETosis upon contact with neutrophils and were used to induce local NETs formation.[33] 50 µL of a 20 mg/mL suspension of MSU crystals in PBS was injected subcutaneously into the left hind paw, between the metatarsals 2 and 3. As a control, the right paw was injected with saline which did not result in any swelling and hence no gout attack. Measurement of paw thickness was then conducted using an electronic caliper.

The animal imaging was performed using Li-COR Pearl Trilogy In-vivo imager (LI-COR Biosciences GmbH, Germany). Excitation was performed with 685 and 785 nm lasers and emission was analyzed at 720 and 820 nm channels, respectively using a 85 µm resolution. Images were normalized using native Image Studio software, provided by the device manufacturer and all measurement parameters were strictly controlled to be identical withing measurement series, as represented in each image.

In the pre-test experiment conjugate **4e** was injected at 20 nM per mice i.p. in the following set-up: a) 24h before measurement; b) 24h and 6 h before measurement; c) 6h before measurement. Signal ratio of treated and untread footpad (+/- MSU) were then compared and injection 6h before imaging was selected as providing the strongest signal. Injection dose was increased to 30 nM per mice.

Then, conjugate **4e** (30 nM per mice) was injected i.p. into 5 sibling mice, 18h after MSU injection. 6h post **4e** injection (and 24 h post MSU injection) mice were imaged for NIR fluorescence at ex. 685 and em. 720nm. Fluorescence of the compound at ex. 785 nm and em 820 nm was minimal and was ignored. The data obtained are provided in Figures 6C, D, E, main text.

Characterization data for new conjugates

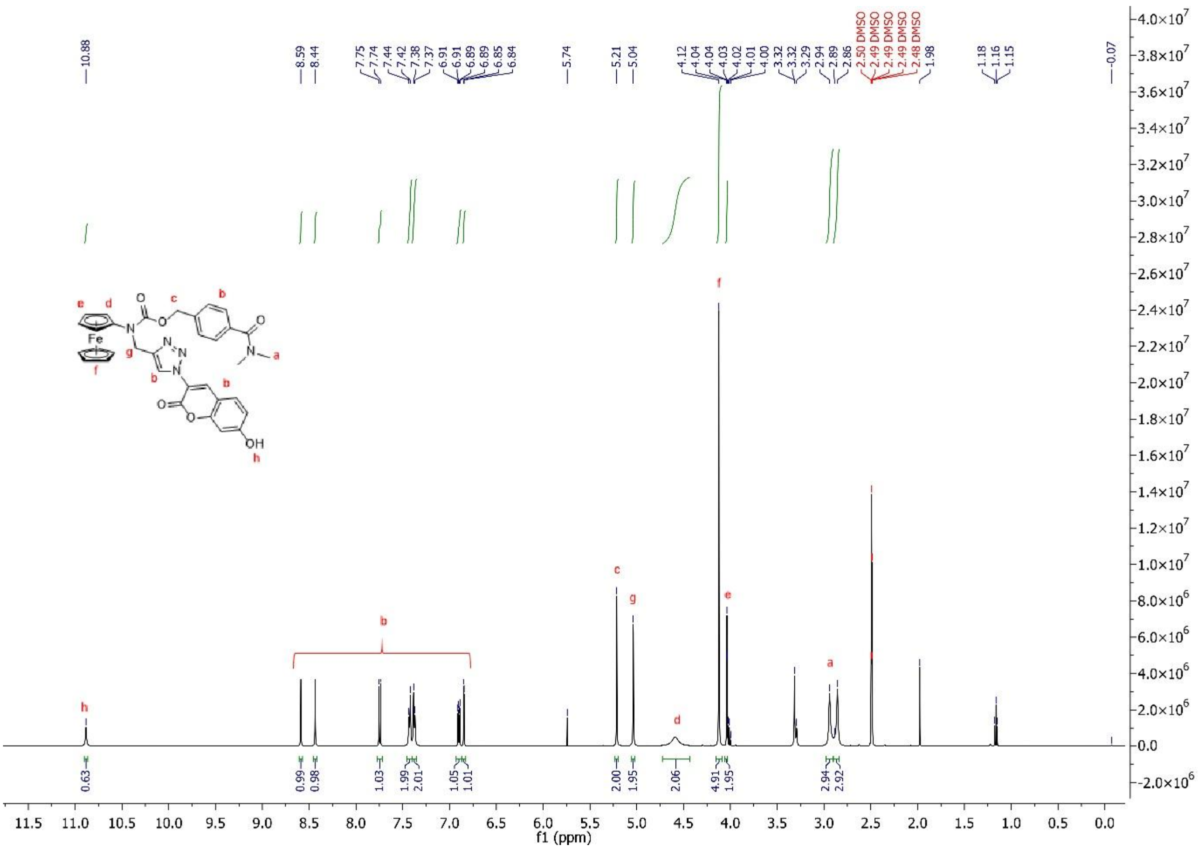

Figure S5. ^{1}H-NMR spectrum of conjugate **4b** in DMSO-d_6.

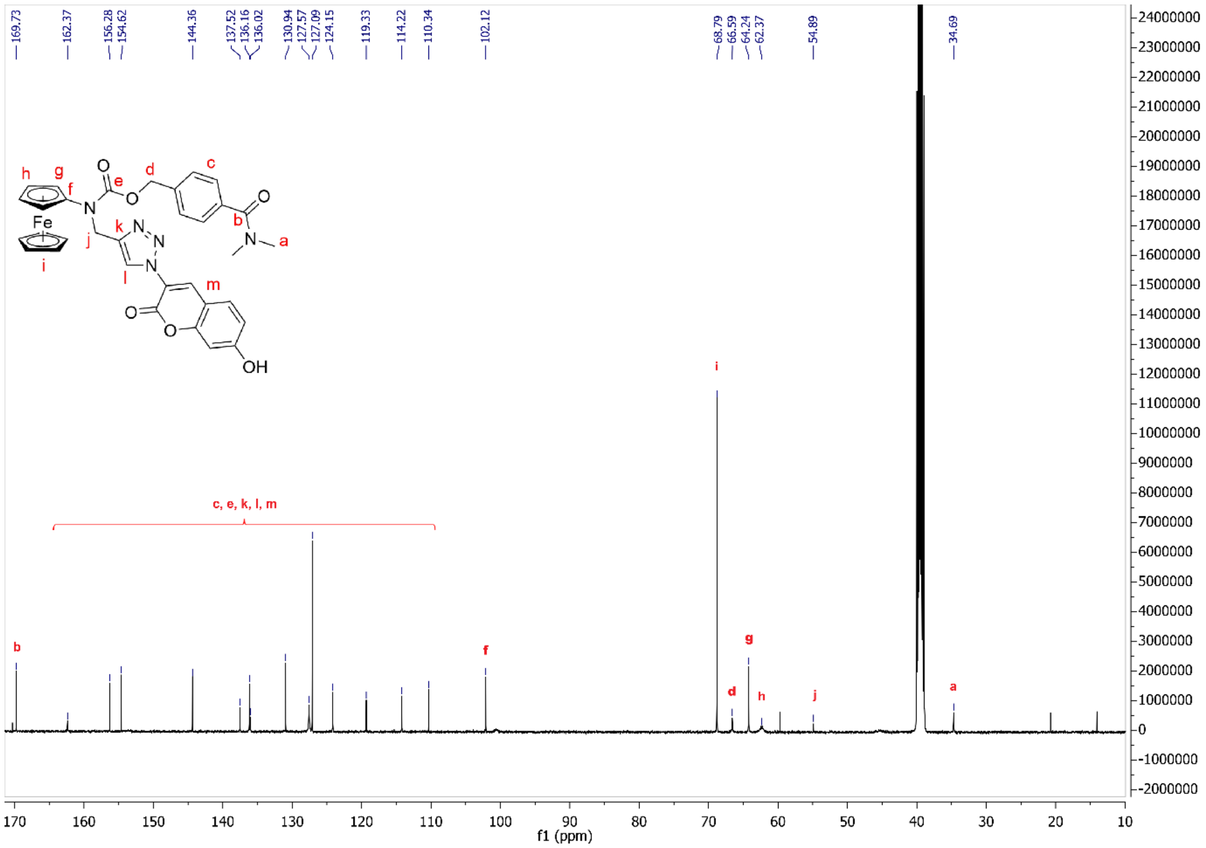

Figure S6. ^{13}C-NMR spectrum of conjugate **4b** in DMSO-d_6.

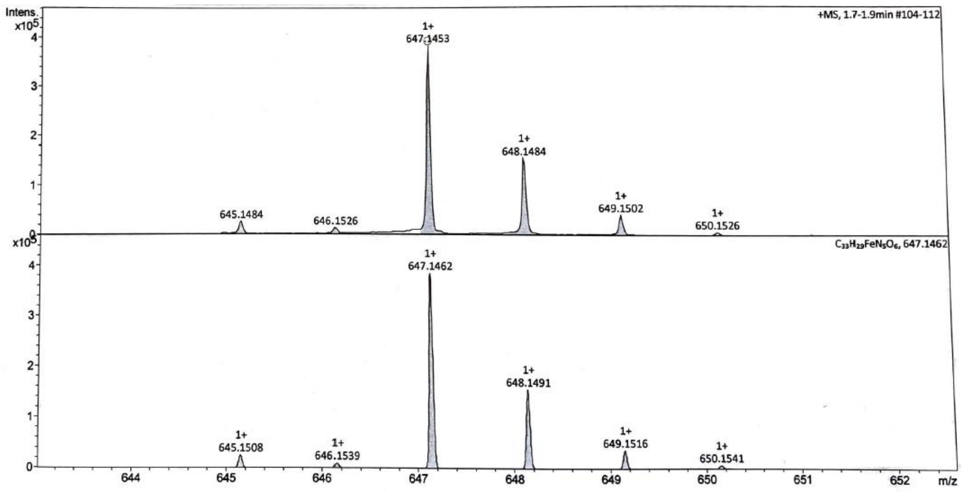

Figure S7. ESI mass spectrum of conjugate **4b**: upper plot – experimental spectrum; bottom plot – theoretical spectrum.

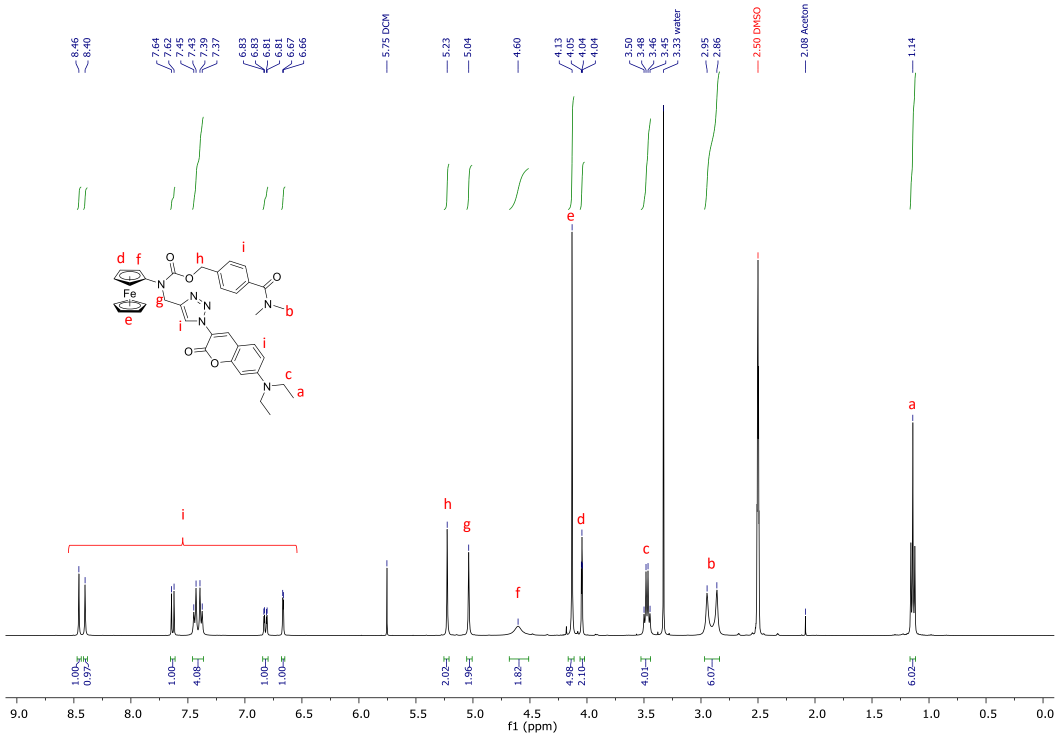

Figure S8. ^{1}H-NMR spectrum of conjugate **4d** in DMSO-d_6.

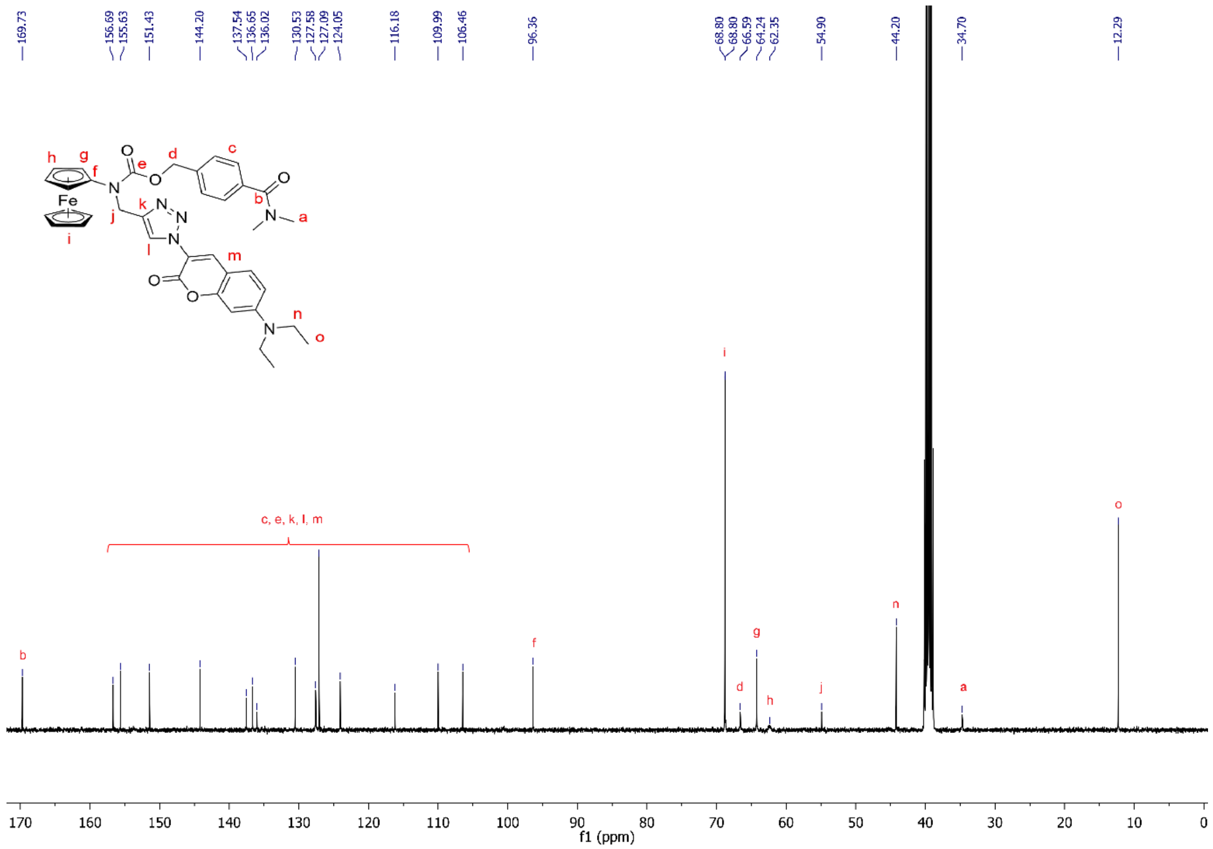

Figure S9. ^{13}C-NMR spectrum of conjugate **4d** in DMSO-d_6.

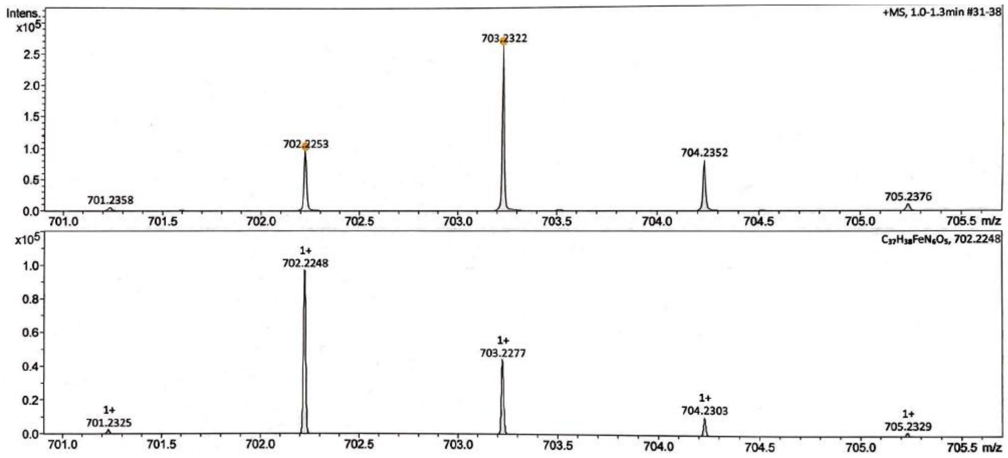

Figure S10. APPI mass spectrum of conjugate **4d**: upper plot – experimental spectrum; bottom plot – theoretical spectrum.

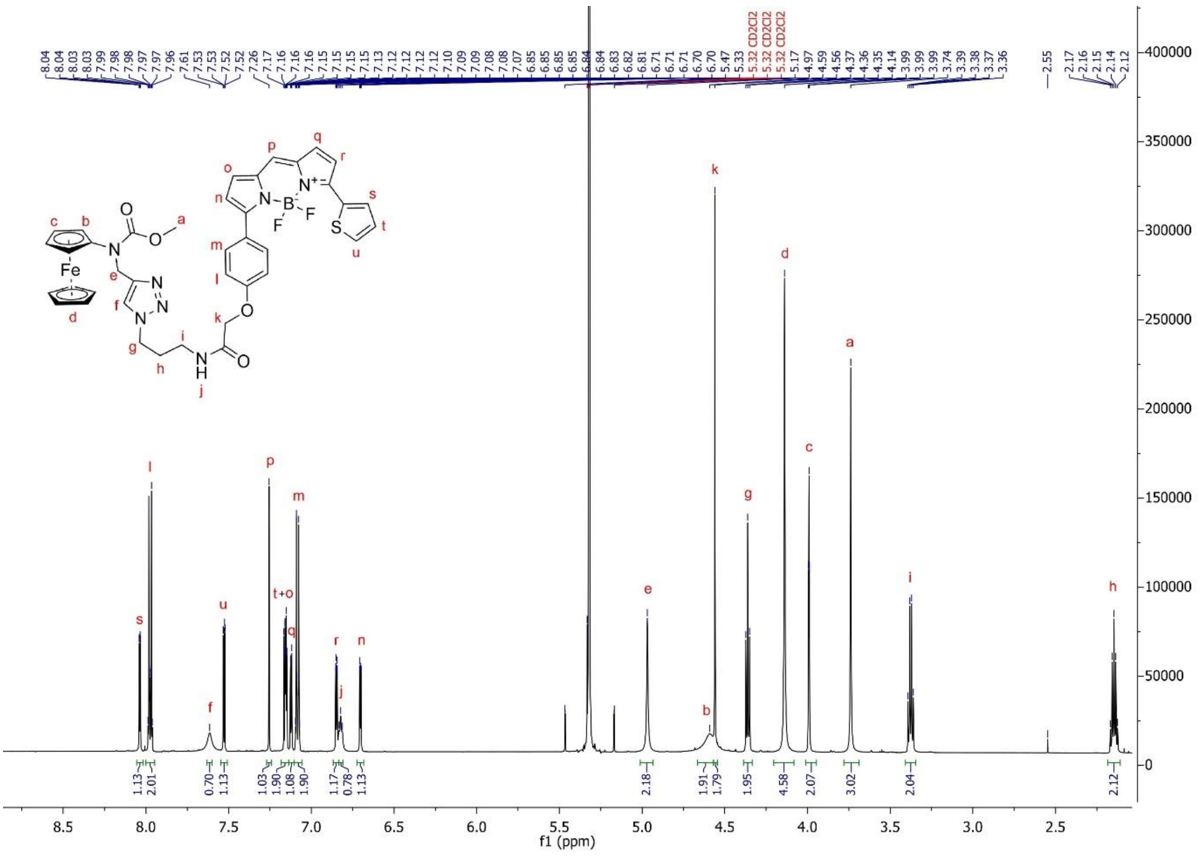

Figure S11. ^{1}H-NMR spectrum of **4e** in CD_2Cl_2.

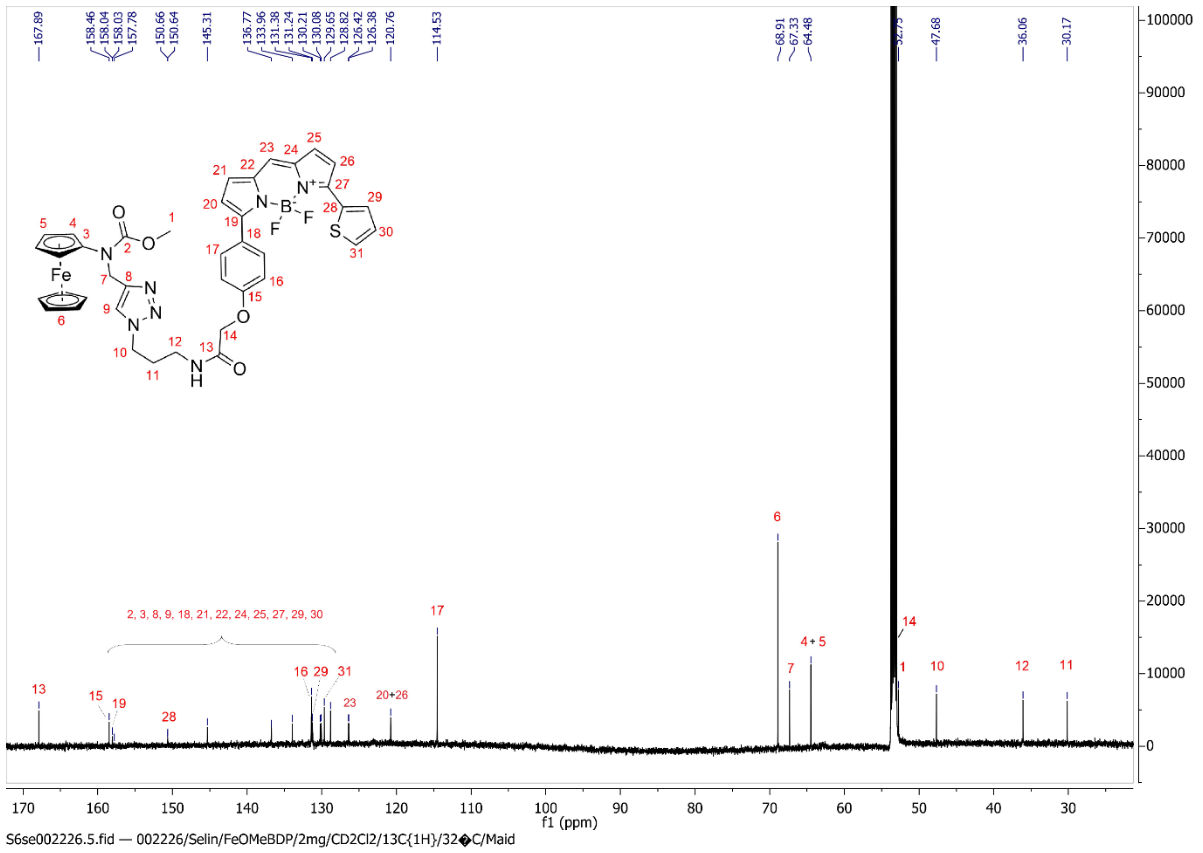

Figure S12. ^{13}C-NMR spectrum of **4e** in CD_2Cl_2.

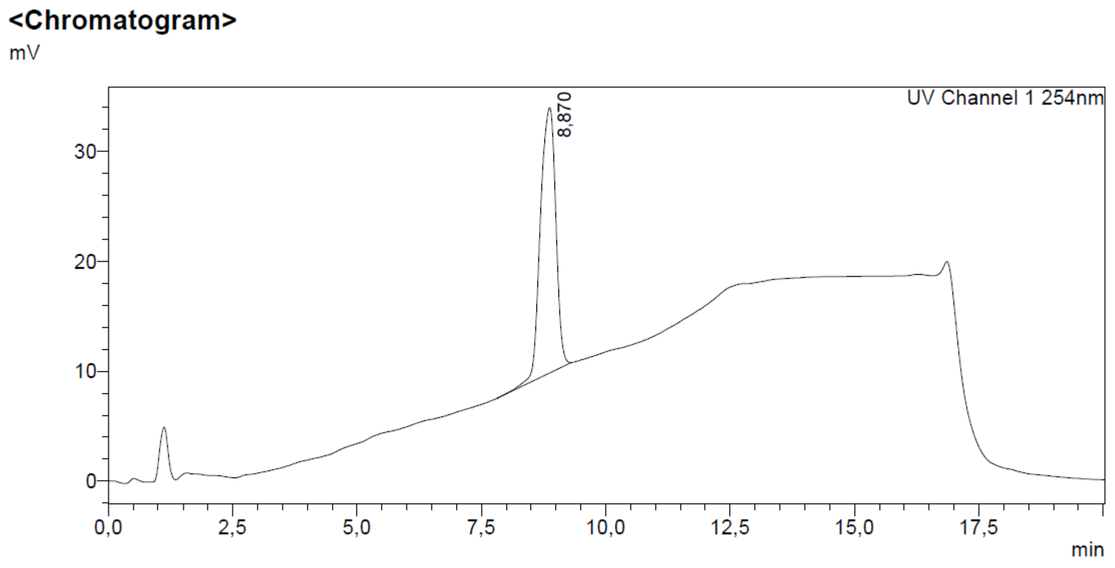

Peak Table

UV Channel 1 254nm

Peak#	Ret. Time	Area%
1	8,870	100,000
Total		100,000

Figure S13. HPLC-UV (fraction monitoring at 254 nm) trace of conjugate **4e**.

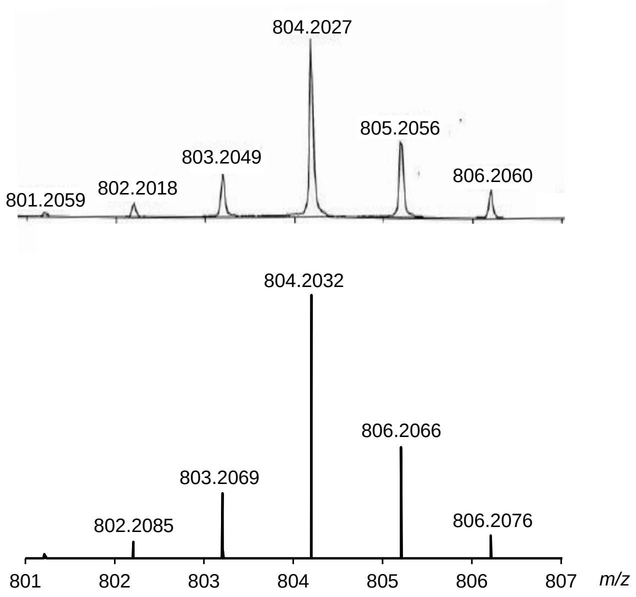

Figure S14. APPI mass spectrum of conjugate **4e**: upper plot – experimental spectrum; bottom plot – theoretical spectrum.

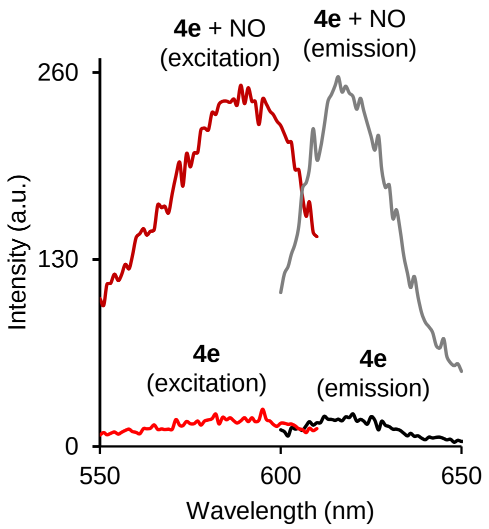

Figure S15. Excitation spectra (emission at 620 nm) and emission spectra (excitation at 590 nm) of conjugate **4e** (100 nM) in triethylammonium acetate buffer (5 mM, pH 7.4) containing 20% CH_3CN (v/v) in the presence (“+NO”) or absence of DEA NONOate (1 mM, incubation time 75 min).

References

[32] K. Sivakumar, F. Xie, B. M. Cash, S. Long, H. N. Barnhill, Q. Wang, "A Fluorogenic 1,3-Dipolar Cycloaddition Reaction of 3-Azidocoumarins and Acetylenes" *Org. Lett.* **2004**, *6*, 4603.

[33] S. K. Mavileti, G. Bila, V. Utka, R. Bilyy Jr., E. Bila, E. Butoi, S. Gupta, P. Balyan, R. Bilyy, S. S. Pandey, “Squaraine-peptide conjugates as efficient reporters of neutrophil extracellular traps-mediated chronic inflammation” *ACS Appl. Mat. Int.* **2025**, *17*, 9140.
